# Corporate Accountability Towards Species Extinction Protection: Insights from Ecologically Forward-Thinking Companies

**DOI:** 10.1007/s10551-021-04800-9

**Published:** 2021-04-10

**Authors:** Lee Roberts, Monomita Nandy, Abeer Hassan, Suman Lodh, Ahmed A. Elamer

**Affiliations:** 1 Brunel University London, Uxbridge, UB8 3PH UK; 2 University of the West of Scotland, G226, Gardner Building, Paisley, PA1 2BE UK; 3 Middlesex University, The Burroughs, Hendon, London, NW4 4BT UK

**Keywords:** Biodiversity, Species extinction, Deep ecology, Legitimacy, Poisson pseudo-maximum likelihood, Stakeholders

## Abstract

This paper contributes to biodiversity and species extinction literature by examining the relationship between corporate accountability in terms of species protection and factors affecting such accountability from forward-thinking companies. We use triangulation of theories, namely deep ecology, legitimacy, and we introduce a new perspective to the stakeholder theory that considers species as a ‘stakeholder’. Using Poisson pseudo-maximum likelihood (PPML) regression, we examine a sample of 200 Fortune Global companies over 3 years. Our results indicate significant positive relations between ecologically conscious companies that are accountable for the protection of biodiversity and species extinction and external assurance, environmental performance, partnerships with socially responsible organizations and awards for sustainable activities. Our empirical results appear to be robust in controlling for possible endogeneities. Our findings contribute to the discussion on the concern of species loss and habitat destruction in the context of corporate accountability, especially in responding to the sixth mass extinction event and COVID-19 crisis. Our results can also guide the policymakers and stakeholders of the financial market in better decision making.

## Introduction

In recent years, policymakers, NGOs, academics, and companies have devoted greater attention to the strategic implications of biodiversity loss and species extinction (hereafter B/E). Yet, the relationship between corporate accountability^1^ in terms of species^2^ protection and factors affecting such accountability (assurance, environmental performance, country, partnerships, etc.) remains underexplored in accounting and business literature (Atkins & Atkins, 2018; Gaia & Jones, 2019; Reade et al., 2015). Biodiversity and species extinction are part of wider global environmental challenges facing humanity (Sobkowiak et al., 2020), with the United Nations Sustainable Development Goals (SDGs), specifically SDGs 14 and 15^3^ the most recent call to action to develop solutions and protect the planet from further biodiversity loss^4^ (UN, 2020a).

It is acknowledged that business activities are recognized as one of the main drivers of biodiversity loss and species extinction (Hassan et al., 2020a; Maroun & Atkins, 2018; Roberts et al., 2020a). Thus, we contribute to the extant literature and examine how the accountability of ecologically conscious/forward-thinking^5^ (hereafter EC/FT) companies can prevent further species and biodiversity loss in the future and align with SDGs 14 and 15. Our motivation for this paper is to respond to the recent calls to contribute to developing solutions for the B/E crisis (Gibassier et al., 2020). This research is significantly timely, as experts suggest that pandemics such as COVID-19 are a result of habitat loss, wildlife trafficking, and humanity’s destruction of biodiversity (Ceballos et al., 2020; Johnson et al., 2020). Potentially, COVID-19 may not be an isolated pandemic; therefore, there must be a seismic shift from companies in valuing and protecting nature. In addition, biodiversity loss is now recognized as one of the top five global risks (WEF, 2020)^6^ with severe implications for society if transformational changes are not made (WHO, 2020).

The above discussion motivates us to examine how EC/FT companies that initiated efforts in conserving and protecting species before the current pandemic can influence companies’ reporting in the post-COVID-19 era. Therefore, we would expect corporates to consciously make tremendous efforts in conserving biodiversity and protecting species from extinction as companies realize their dependence on nature. Thus, our findings can guide and encourage companies to improve future reporting to achieve SDGs 14 and 15 targets by 2030, as “accounting academics can and should play a substantial role helping embed policy and action at an organizatinal level in a way that contributes towards the achievement of the SDGs” (Bebbington & Unerman, 2018, p. 2). The findings of this study will extend the existing academic findings on emerging issues about B/E accounting (e.g., Adler et al., 2018; Atkins & Maroun, 2018; Hassan et al., 2020a, b). The combination of the theoretical framework along with robust empirical findings will assist academics in advancing this stream of emerging literature, and help companies to understand how to consider various factors to be sustainable in the future. Additionally, this will give a clear indication to regulators about the changes required to motivate companies for preventing further B/E loss.

Furthermore, extant B/E literature is limited. Specifically, most studies employed one theoretical construct to explain the companies’ accountability towards the extinction of species, which contains several caveats. We respond to the previous call (Gaia & Jones, 2019) that a single theory is not adequate in explaining B/E disclosure, by adopting a triangulation of deep ecology, legitimacy, and stakeholder theories. By applying deep ecology and stakeholder concepts, we support the argument of Roberts et al. (2020b), who suggest that species are of fundamental value to business survival and are main stakeholders in society; therefore, companies should consider species as an important stakeholder of their business. Legitimacy theory can explain how a company’s legitimacy can be achieved by considering species as important as other stakeholders in company operations. The comprehensive theoretical model allows us to contribute to the limitation of the extant literature theoretically. To date, seminal contributions in literature stimulate the development of species protection by providing extinction accounting frameworks (Atkins & Maroun, 2018; Hassan et al., 2020b) and examination of organizational accountability for biodiversity (Adler et al., 2018; Maroun et al., 2018). Emerging studies (e.g., Raar et al., 2020) open a debate on how the incorporation of biodiversity accountability can assist in the prevention of further species loss. Flowing from extant literature, this paper builds on these bodies of work to empirically examine company disclosure on species and what factors motivate relationships for such disclosure. This research responds to Roberts et al. (2020b) who identify the urgency in B/E literature for the examination of species protection disclosure to extend knowledge in the field and contribute to advancing solutions.

Based on the above discussion, the objective of this paper is to investigate factors affecting the relationship between corporate accountability and species protection of EC/FT companies. We believe that providing disclosure on species protection enhances stakeholders’ trust and accountability (Hassan et al., 2020b). To empirically test our idea, this study uses a sample of the top 200 companies from Fortune Global in 2012, 2014, and 2016, to highlight how EC/FT companies reported on the protection of species before the recent pandemic and provide a springboard to develop solutions after the pandemic. These companies directly or indirectly make significant use of ecosystems, and therefore gain the most public attention (Adler et al., 2018; Hassan et al., 2020b). Our justification is to present how EC/FT companies report on conserving and protecting biodiversity and species, which could influence reporting in the recent and coming years. By applying advanced empirical analysis, we find that companies that are disclosing responsibly about species and are actively protecting against extinction, for which these companies are recognized by awards, have extended partnerships with species protection organizations and are also getting favorable assurance for their business.

The study makes several contributions to the extant B/E literature. First, to the best of our knowledge, no studies have examined the relationship between species disclosed by organizations and its determinant factors. Therefore, this paper aims to close this gap by contributing to the extant B/E literature and demonstrate how EC/FT companies are displaying significant efforts on species protection and restoring habitats. Second, theoretically, we adopt a triangulation of theories; deep ecology, legitimacy and stakeholder theories. We also suggest that species should be considered as one of the main stakeholders’ categories in addition to customers, employees, etc., as businesses have a two-way relationship with biodiversity and species, including both the impact of companies on biodiversity and the impact of biodiversity on companies (Adler et al., 2018). Third, our paper has an empirical contribution. Instead of adopting OLS regression that is commonly used by previous studies (Adler et al., 2018; Hassan et al., 2020b), we test and create Poisson pseudo-maximum likelihood (PPML) regression with a multi-way fixed effects model. We believe the PPML model is more relevant to explore the relationship between the species disclosed by companies and determinant factors because our disclosure index includes positive count values only. Fourth, our study has a number of implications for policymakers. The proposed empirical model and theoretical framework in this study will allow policymakers to process a post-2020 framework to reshape the global relation with nature (UN, 2020b) and support the United initiatives and achieve the SDGs 14 and 15 objectives. The study will guide decision-makers to understand how disclosure of species protection by companies can assist society to mitigate further B/E loss in the future. Finally, our paper responds to recent calls (Gaia & Jones, 2019; Lambooy et al., 2018) to develop solutions by showing how the potential for reporting for B/E can mitigate the further risk of ecological collapse.

The rest of the paper is structured as follows: the next section covers the literature review. Section three discusses the theoretical literature. Section four discusses empirical literature and hypothesis development. Section five illustrates the research design. Section six presents the empirical analysis. Finally, a discussion of findings and limitations is exhibited in the last section.

## Corporate Accountability, Ecology, and Species Extinction Protection Around the World

Research on corporate accountability for B/E is in its infancy (Addison et al., 2019; Adler et al., 2017) and is an emerging strand of literature (Haque & Jones, 2020). To date, academics have been relatively silent on examining the role of companies in conserving biodiversity and species protection (Adler et al., 2018) and thus, there is a huge call for company awareness from stakeholders of businesses (Hassan et al., 2020b). To provide context, B/E accounting emerges from biodiversity reporting and this extends to include the ‘extinction’ element due to the severity of the decline of nature (Atkins & Maroun, 2018). The early seeds of biodiversity reporting were set by Jones (1996) who suggested that organizations are stewards of natural capital and have a moral duty to protect and to publicly disclose their efforts (Atkins et al., 2014). Early empirical studies began to explore reporting of companies on biodiversity issues at country level (Boiral, 2016; Rimmel & Jonäll, 2013; van Liempd & Busch, 2013) and industry level (Adler et al., 2017; Boiral & Heras-Saizarbitoria, 2017). Prior studies are consistent in finding that most companies provide poor disclosure, vague statements, and are generally unimpressive with their efforts to manage and protect biodiversity. Literature suggests that companies fail to recognize the risk of biodiversity loss, leaving them unconvinced that conservation warrants their efforts (Skouloudis et al., 2019). It seems to be only when endeavors provide rewards of stakeholder impressions or reputational advantage that companies will pay attention (Bhattacharya & Managi, 2013; Hassan et al., 2020b).

It is argued that biodiversity reporting on its own is insufficient (Atkins & Maroun, 2018; King & Atkins, 2016). The widely adopted Global Reporting Initiative (GRI) standards only make some reference to biodiversity (Jones & Solomon, 2013), and therefore company accountability is limited. Companies following the GRI framework are found to be indulging in impression management (Gray & Milne, 2018; Solomon et al., 2013), with a lack of consistent, transparent reporting. Atkins and Atkins (2018) suggest that continuing to report in this method will lead to a fossil record of species. Haque and Jones (2020) support the previous arguments and mention that companies are using GRI standards as a mechanism to refer to biodiversity, providing symbolic statements and failing to provide clarity on operational impact to natural capital.

In response to the limitation mentioned above, extinction accounting evolves from biodiversity reporting by critically recognizing the need to address the current extinction crisis (Atkins & Maroun, 2018). Incorporating GRI biodiversity principles, extinction accounting aims to promote change by companies and reverse species loss (Atkins & Atkins, 2018; King & Atkins, 2016), providing companies opportunity to disclose how they are acting to prevent further extinctions (Hassan et al., 2020b).

The framework provides the opportunity to include narrative detail and self-reflection, and to further articulate extinction prevention measures with the hope that this will lead to changes in company behavior (Hassan et al., 2020b). Furthermore, companies should seek to consolidate numerical data and narrative and pictorial evidence to comprehensively communicate conservation efforts (Atkins & Maroun, 2020). Atkins (2020) argues that companies should be integrating extinction accounting into annual reports to trigger change and would have to “assess the populations of threatened species living near their operations; work out whether their business puts them at risk; come up with plans to protect them; and explain them to investors”. Studies from extinction accounting give evidence of a genuine concern for nature and represent the need for compassion for species (Atkins et al., 2018; Buchling & Maroun, 2018); they support the idea that companies begin to acknowledge their dependence on natural capital. Motivated by extinction concern, evidence of species names emerges in corporate reports (Adler et al., 2018; Atkins et al., 2014). However, an argument builds that certain alluring or desirable species may attract more attention from companies than others, as Weir (2018) observed that mammals and birds warranted greater conservation efforts than insects and invertebrates. Atkins et al. (2014) reinforce this theory, reporting that favoritism may be shown towards certain species, especially species which are beneficial to humans.

Smith et al. (2019) found that the corporate motivation to engage in conservation efforts is unclear. However, the existing studies assist companies to recognize that the threat of extinction is a material risk (Addison et al., 2019). Global policymakers are also taking the initiative to encourage companies to be accountable towards the extinction of species. For example, The Natural Capital Coalition (2020) urges companies to realize that their success is driven by ecosystems; the loss of any species to extinction is devastating, but species which provide such intrinsic worth to industries is frightening.^7^ Indeed, the global Aichi targets of the ‘Strategic Plan for Biodiversity 2011–2020’ have been considered a failure as biodiversity decline is accelerating at an unprecedented rate, with humanity’s legacy of biodiversity at a crossroads for future generations (CBD, 2020). The most recent call to action is the United Nations SDG targets ‘The 2030 Agenda for Sustainable Development’. Global governments have agreed on a vision of ‘Living in harmony with nature’ by 2050, and it is imperative lessons are learned from the past decade if the SDGs are to be met. The UN Biodiversity Conference in September 2020 emphasizes the need for “Urgent action on biodiversity across all sectors and from all actors” to meet the SDG target of 2030 (CBD, 2020). Furthermore, the post-2020 biodiversity initiatives must be achieved, or humanity could potentially face future pandemics.

The current COVID-19 crisis provides forewarning to further encroachment between natural capital and humans that can have detrimental impacts (Carrington, 2020). Corporates must make transformational changes to restore their relationship with nature and engage in sincere stewardship of natural capital, as it is their moral duty to protect future generations (Gaia & Jones, 2019). Turning a blind eye to warning signs could be catastrophic for business prosperity and survival. There is no extensive literature to explain the accountability of companies towards species extinction. Therefore, in this study, we discuss how the disclosure of species by global companies can assist them in achieving environmental awards, gain assurance from Big 4,^8^ and allow companies to expand biodiversity partnerships leading to a financially sustainable company. The findings of this study will be an example for stakeholders of companies and will encourage academics to extend the studies on B/E to assist companies to be more accountable towards B/E. Furthermore, to meet SDGs 14 and 15, we offer a solution that can assist in preventing further decline by advocating that companies transform their reporting practice to include information on their efforts to protect biodiversity and species.

## Theoretical Literature Review

Prior B/E research applies various theories to explain company viewpoints including impression management (Boiral, 2016), greenwashing (Hassan et al., 2020b), and legitimacy (Adler et al., 2017; Bhattacharyya & Yang, 2019). However, we observe that companies have not done enough to protect species from extinction, and, as experts explain, it may be that this is one of the reasons for zoonotic disease spillover such as COVID-19 (Ceballos et al., 2020). Lack of a comprehensive theoretical model cannot motivate companies to identify a strategy for species protection. Therefore, we introduce a triangulation of theories, namely deep ecology, legitimacy, and stakeholder theories. Deep ecologists are of the view that nature has intrinsic value, and all nonhuman life should be preserved (Naess, 1989, 2008), thus rejecting anthropocentric shallow ecology, which places humans of most importance, believing nature has value because of what it contributes to human satisfaction (Callicot, 1990, 1994; Thompson & Barton, 1994). Deep-ecologists debate whether environmental and extinction crises are human-induced (Samkin et al., 2014) and support the suggestion that the crisis is the result of dominating anthropocentric bias in corporate behavior (Atkins et al., 2014). Absolute deep ecology would reject business use of natural assets as a commodity. However, to mitigate corporate financial risk and prevent societal collapse, a middle ground must be reached. Companies must consider embedding an ecological culture and stewardship (Jones, 1996) by protecting and investing in nature. For example, Gaia and Jones (2019) found that elements of deep ecology must be ethically rooted to enable a sustainable society. Similarly, Samkin et al. (2014) found deep ecology embedded in biodiversity disclosures and mention that the approach requires a long-term commitment. Embracing deep ecology is not to put a financial value on species as this would be refuted by deep-ecologists; rather, companies must evaluate their behavior and engage in a balanced perspective to mitigate the risk of financial loss, together with responsibly protecting species and habitats. The relationship with nature must be realigned and eliminate the arrogant profit-seeking objectives, which have driven us to a planetary emergency (Gray & Milne, 2018).

Empirical evidence considers corporates to be rife in legitimizing activities (Boiral & Heras-Saizarbitoria, 2017; Cho & Patten, 2007; Milne & Gray, 2013; Patten, 2002). Extant literature considers B/E accounting as a continuation of corporate social responsibility (CSR) research into corporate disclosure practices (Bebbington & Larrinaga, 2014; Hassan et al., 2020b). Legitimacy theory is one of the most applied theories to explain the increasing CSR reporting over the past two decades (Hassan & Guo, 2017) and it “involves the selective disclosure of positive actions resulting in misleading and biased reporting” (Mahoney et al., 2013, p. 352). Extant literature uses legitimacy theory to explain that dishonest companies misreport their CSR efforts to capitalize on their face value to influence stakeholders’ perceptions and gain legitimacy (Lyon & Maxwell, 2011; Zijl et al., 2017).

Adler et al. (2018) argue that biodiversity and threatened species information is provided in order to fulfil the desires and expectations of stakeholders. Bhattacharyya and Yang (2019) specifically note that considering the current planetary emergency, for businesses to gain societal legitimacy, they must increase biodiversity disclosure. Lewis (2016) explains that legitimacy is a practice that is deceivingly used to endorse that organizations’ policies or practices are environmentally friendly, when arguably they are not. Patten (2015), for example, explains that an organization with specific environmental adversity intensifies the extent of CSR reporting to signal to stakeholders that the organization is addressing the concern. This continues a tradition of impression management-oriented literature in environmental accounting (Hassan et al., 2020a, b). This research argues that companies provide disclosure to legitimize companies’ concerns for B/E issues (Adler et al., 2018; Cho et al., 2015a; Lyon & Maxwell, 2011).

As the species extinction crisis intensifies, it can be expected that companies must meet external pressures (Rimmel & Jonäll, 2013) and enhance reputation to maintain their “licence to operate” (Adler et al., 2017, p. 1714). Research highlights that stakeholder and legitimacy theories overlap in social and environmental studies (Deegan, 2002; Gaia & Jones, 2017, 2019). Stakeholder theory has been used to explain the needs and expectations of human groups and individuals affected by the company (Boiral & Heras-Saizarbitoria, 2017; Gaia & Jones, 2019). Theoretically, we recognize a limitation within stakeholder literature that prior studies have failed to recognize or explain for the prevention of further species loss. Roberts et al. (2020b) argue that species should be included as a main stakeholder with the established groups of employees, NGOs, government agencies, environmental groups, and customers (Jones, 1995; Schaltegger et al., 2017). We believe that for deep ecology to be embedded in corporate strategy, species of flora and fauna should be considered as one of the main stakeholders’ categories, discarding human hierarchy, for companies to protect and restore species and their habitats. In this vein, we focus on a triangulation of deep ecology, legitimacy, and stakeholder theories, which can explain the relationship between species numbers and determinant factors. We recognize that corporates may be disclosing species-specific information for legitimizing purposes, but we are also hopeful that those companies who are disclosing species numbers have started to realize the intrinsic worth of natural capital and are consequently embedding ecological culture and displaying a genuine concern for the extinction crisis by identifying species as stakeholders. We hope that providing disclosure on species protection will be the new norm for other companies to follow. Societal health is underpinned by nature (Roberts et al., 2020a); we expect that by application of the triangulation of theories, we can assist companies to explain the underlying motivation to reform B/E impact, which will reduce potential future pandemics (Ceballos et al., 2020).

## Empirical Literature Review and Hypotheses Development

B/E accounting is considered as an extension of CSR (Bebbington & Larrinaga, 2014; Bhattacharyya & Yang, 2019; Hassan et al., 2020b). From the existing literature, we identified that the assurance by leading assurance providers, presence of partnerships, environmental performance, and environmental award are some important factors that will assist the stakeholders to assess the accountability of the companies towards nature. In the following section, we discuss these factors in detail to develop the hypotheses related to the research question, which is supported by the comprehensive theoretical model explained previously.

### Species and Assurance

Sustainability reporting is established in mainstream practice to meet the needs of societal expectations (Junior et al., 2014; Kolk & Perego, 2010). However, it is argued in the literature that the reliability and quality of information falls short (Cho et al., 2015a). Boiral et al. (2018) imply that information can be biased and reporting by companies is used as a window-dressing activity (Boiral, 2016), and thus, the credibility of information is questioned (Gray, 2010). To enhance the quality and credibility of sustainable reporting in literature, we find that companies prefer to use third-party assurance from accounting firms (Maroun, 2018). Involvement of third parties can increase the confidence of the stakeholders in disclosure (Simnettt et al., 2009). Human stakeholders perceive the professionally audited reports from the external assurance provider as a confident and legitimate report of the company performance (Cho et al., 2015a).

Because of the immense presence of independence in the report prepared by the external assurance provider, companies who are under public pressure due to poor performance may prefer to seek external independent assurance to indicate a better performance to the stakeholders (Boiral et al., 2018; Maroun, 2018). But external assurance is also required to identify which companies are better in addressing the B/E risk, which is prominent (IPBES, 2019) and related to human behavior. The external assurance report can help the stakeholders to understand how the companies are following deep ecology principles by engaging in the stewardship of natural capital (Bhattacharyya & Yang, 2019). Legitimacy theory has been dominant in the social and environmental accounting literature (Belal & Owen, 2015; Giordano-Spring et al., 2015). Many studies (e.g., Ball & Craig, 2010; Cho, 2009; Cho & Patten, 2007; Hassan & Guo, 2017; Patten, 2015; Tilling & Tilt, 2010) empirically examine legitimacy theory and support the argument that companies voluntarily provide environmental information to gain legitimacy. The theory also predicts that organizations who are more likely to be subject to public pressure and legitimacy threats due to negative CSR performance may hire third parties to provide assurance to indicate favorable performance (Boiral, 2016; Cho et al., 2014; Maroun, 2018). Consequently, independent third-party assurance can help to deflect attention from negative B/E performance, lessen legitimacy risks, and install improved confidence among stakeholders (Gürtürk & Hahn, 2016; Perego & Kolk, 2012). More specifically, these firms actively hire third parties that provide limited assurance to portray that the B/E information released in CSR reports is credible, in order to improve stakeholders’ confidence and enhance corporate reputation and perceived legitimacy (Cho et al., 2014; Odriozola & Baraibar-Diez, 2017). Nevertheless, as companies must comply with reporting standards, they may prefer the ‘low-quality assurance’ with less scrutiny, and thus they have opportunity to dissociate their revealed from their actual performance.

However, it is also evident in the literature that assurance providers deliver cautious rhetoric, failing to explicitly address issues around the report on biodiversity (Boiral et al., 2019). Companies may prefer to buy less scrutinized ‘low-quality’ assurance options to deflect from their poor performance (Hassan et al., 2020b), and are expected to select limited assurance (Braam & Peeters, 2018) outside of the accounting profession to focus on selected sections of the performance. In post-pandemic reporting, we expect companies to engage in stewardship of protecting species and habitats, embedding a deep-ecological culture by regarding species as a main stakeholder. In the future, assurance providers will scrutinize the company impact on biodiversity. Our motivation for this hypothesis is to extend existing literature which finds a positive relation between B/E disclosure and external assurance (Hassan et al., 2020b). Therefore, we examine whether external assurance is an influencing factor in protecting species. By applying deep ecology and stakeholder theory, companies are expected to commit to protecting species (Samkin et al., 2014) and valuing them as stakeholders. The contradictory findings of the importance of external assurance motivated us to examine the influence of external assurance on companies’ accountability towards the extinction of species. Thus, we propose the following hypothesis:

#### Hypothesis 1

There is a positive relationship between the number of species and buying assurance from an external assurance provider.

### Species and Environmental Performance

Legitimacy theory helps to explain why companies with poor environmental performance have higher environmental scores (Mahoney et al., 2013). In the literature, we find that the more extensive disclosure companies provide, the more the firm’s reputation is enhanced (Cho & Patten, 2007; Cho et al., 2012). Poor performers are anticipated to disclose in order to gain legitimacy and enhance societal perception (Clarkson et al., 2008). Hassan et al. (2020b) classify companies into poor and better performers and find companies with poor environmental performance disclose more and offer justification to defend legitimacy. Based on this argument, we expect that poor performers will disclose more species information to maintain legitimacy, and as a result, the companies can be more accountable in mitigating the extinction of species. If species are accepted as one of the main stakeholders, we expect EC/FT companies to propose long-term value creation and suppress demand on natural capital, committing to achieving SDGs 14 and 15. Our motivation is to extend B/E literature (Hassan et al., 2020b) and contribute to it by empirically examining, in support with legitimacy theory, the relationship between species and poor environmental performers. Thus, we propose the following hypothesis:

#### Hypothesis 2

There is a positive relationship between the number of species and poor performers.

### Species and Environmental Award

Gaining an environmental award is an excellent way of displaying positive practices to stakeholders (Cho et al., 2015b; Deegan, 2002), and motivates other companies to disclose CSR practices (Hassan & Ibrahim, 2012). Acquiring awards can show the true commitment of the company to natural capital and confirm their deep ecological perspective (Clarkson et al., 2008). Such awards influence investors to make a positive decision about the company, which leads to favorable future financial performance (Clarkson et al., 2011). Prior B/E literature suggests that companies should report on prizes and awards relating to conservation efforts (van Liempd & Busch, 2013). Awards provide an opportunity to signal genuine concern for nature (Adler et al., 2018; Atkins et al., 2014) and showcase efforts. Hassan et al. (2020b) exclusively find a positive association between B/E disclosure and companies gaining environmental awards. Based on this discussion, our motivation is to extend extant literature and expect companies who disclose species to gain environmental awards, and by doing so, deep ecology supports that they have considered species as stakeholders (Roberts et al., 2020b) in their reporting in order to address the extinction crisis responsibly.

#### Hypothesis 3

There is a positive relationship between the number of species and getting an environmental award.

### Species and Partnerships

Prior literature finds a positive relationship between B/E disclosure and partnership engagement (Adler et al., 2018; Boiral & Heras-Saizarbitoria, 2017; Hassan et al., 2020b). Here, partnership refers to when a company discloses a relationship with at least one conservation or wildlife organization. This can be viewed as a display of good corporate practice (Adler et al., 2018) and a means of seeking public trust (Deegan, 2002). Collaborating with organizations such as WWF (World Wildlife Fund) or IUCN (International Union for Conservation of Nature) can help companies to engage in conservation efforts, and companies are therefore more likely to minimize the extinction crisis (Adler et al., 2018). Partnership engagement will motivate companies to consider species as stakeholders (Atkins et al., 2018; Buchling & Maroun, 2018; Zhao & Atkins, 2018). By supporting this concept, EC/FT companies can commit to achieving SDGs 14 and 15 and can align with long-term value creation. Thus, our motivation is to enhance B/E literature (Adler et al., 2018; Boiral & Heras-Saizarbitoria, 2017) by empirically examining the relationship with species and partnership engagement. Theoretically, deep ecology perspective and regarding species as stakeholders, demonstrates that companies are preserving nature (Atkins et al., 2018) and showing genuine concern for the B/E crises. Thus, we expect that companies which engage in partnerships will disclose more species.

#### Hypothesis 4

There is a positive relationship between the number of species and companies who engage in partnerships.

## Research Design

### Sample Selection

The sample for this research consists of the top 200 companies from the Fortune Global list of 2016. Purposefully, we considered these companies as they are typically leaders in CSR (KPMG, 2017), make significant use of ecosystems, and therefore gain the most public attention (Adler et al., 2018; Hassan et al., 2020b). These companies represent a variety of industries exposed to different levels of biodiversity risk (Addison et al., 2019; Bhattacharya & Managi, 2013) from diverse geographic locations (Hassan et al., 2020b). The top 200 companies are selected from the Fortune Global 500 list as literature supports that the remaining companies rarely disclose biodiversity information (Adler et al., 2018; Hassan et al., 2020b). The investigation period is 3 years: namely, 2012, 2014, and 2016, as one of the variables, environmental performance, is calculated every 2 years. Corporate annual and sustainability reports are downloaded from company websites. Sustainability reports can be referred to as environmental, corporate social responsibility, citizenship, or such reports. Where these reports are missing, we manually collected relevant information from the annual reports. Following prior studies (Addison et al., 2019; Adler et al., 2017), websites were not included in the search. In total, 600 annual and sustainability reports were downloaded (which are accessible on their corporate web pages). After controlling for an outlier, our final sample comprised of 599 companies. Following prior studies (Adler et al., 2018), we used content analysis, and by searching keywords,^9^ we counted for species information. Keyword search and manual collection are followed in this paper to identify companies from 22 countries^10^ and 19 sectors.^11^

### Research Variables

#### Dependent Variable: Number of Species

The number of species count was comprised of species numbers presented in quantitative terms or by naming in qualitative terms. A manual count of all species disclosed on reports were collected and recorded. The number of species counted was protected or conserved or noted as threatened with extinction by the company. Where companies disclosed a group of species, for example, 10 birds, we counted these as 10 species and so on. In the counting of species, duplicate references were eliminated; in other words, species referred to on more than one occasion were counted once.

### Independent Variables

We used assurance, environmental award, presence of partnerships, and environmental score as independent variables (see Table 1). The environmental score was measured by the environmental well-being score from the Sustainable Society Foundations website (Adler et al., 2018; Hassan et al., 2020b). The scores are available per country every 2 years and following Hassan et al. (2020b), we classified the sample into poor performers (score 0–2.9) and better performers (score 3–5). The sample consisted of 290 poor performers and 309 better performers.

**Table 1:** Research variables

	Definition and coding
Dependent variable	Number of species—Total count of number of species collected from published annual reports
Independent variable	Assurance—has a value of “1” if the company has assurance and a value of “0” if not. Data collected from published annual reports
	Assurance by Big4—Company has a value of “1” if assured by one of the big four accounting firms (KPMG, E&Y, PwC, or Deloitte), and a value of “0” if not. Data collected from published annual reports
	Environmental Award—value of “1” if award is given, value of “0” if not. Data collected from published annual reports
	Environmental Score—Company is given a value of “1” if classified a ‘poor performer’ and a value of “0” if classified a better performer. Data collected from Sustainable Society Foundation (SSF). Score is calculated every 2 years and is used in prior studies (Adler et al., 2018; Hassan et al., 2020a, b)
	Presence of Partnerships—Presence of biodiversity/wildlife partnerships, value of “1” given if one or more present, value of “0” if none. Data collected from published annual reports
Control variables	Country—Value of “1” given if the country is classified as developed and a value of “0” if developing. Data retrieved from United Nations website
	Industry—Company has a value of “1” if classified as red/amber-risk zone, and a value of “0” if classified as a green risk-zone. Classification recommended by F & C Asset Report (2004)
	Governance—Is the average score of voice & accountability, political stability, government effectiveness, regularity quality, rule of law, control of corruption and corruption index taken from Worldbank.org
	GDP growth—(annual%)—World Development Indicator. Data collected from Worldbank.org
	Inflation—GDP deflator (annual%) World Development Indicator. Data collected from Worldbank.org
	Log (Forest area)—Forest areas (sq. km)—World development indicator. Data collected from Worldbank.org
	CO_2_ emission—(metric tonnes per capita)—World Development Indicator. Data collected from Worldbank.org
	Log (Revenue)—Data collected from published annual reports
	Leverage—Total debt/total assets. Data collected from published annual reports
	Firm size—Natural logarithm of total assets. Data collected from published annual reports

#### Control Variables

For this research, we considered leverage, firm size, and revenue as control variables. In addition, we used country-level variables. Following Hassan et al. (2020b), we classified the sample into developing and developed countries, according to the United Nations classification. There are 177 developing and 422 developed firm-year observations in our sample. The industry was controlled in the study and was classified by risk exposure according to the three categories of the F & C Asset Report (2004) risk level (red is high risk; amber is medium risk and green is low risk).^12^ In accordance with Hassan et al. (2020b), we grouped red and amber to “high risk” classification, and green remained “low-risk” classification. The total sample consisted of 219 high-risk firm-year observations and 380 low-risk firm-year observations. Governance indicators are widely used in multi-country studies (Nguyen et al., 2015; Waldron et al., 2017) with empirical evidence finding a positive correlation of company performance with country-level governance (Luo et al., 2012). Therefore, we added seven regularity governance level indicators (see Table 1) developed by Kaufmann et al. (2011) collected from the World Governance Indicator dataset. In addition, we added World Development Indicators, like GDP growth, inflation, the log of forest area, and C0_2_ emissions (Spaiser et al., 2017; Stephan et al., 2015).

## Empirical Results and Discussion

### Summary Statistics

**Fig. 1:**
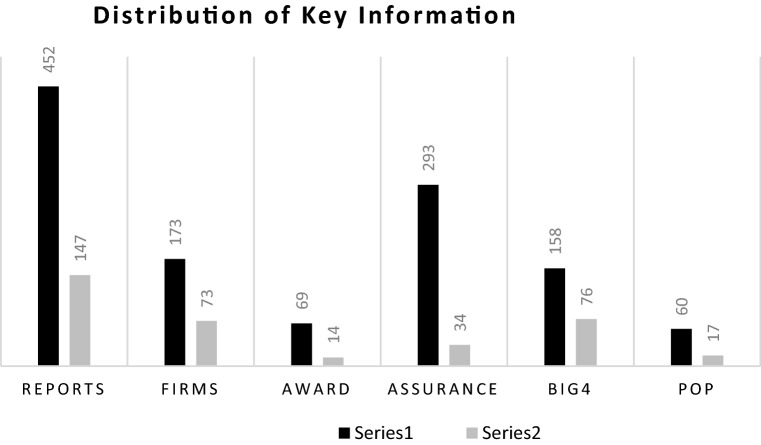
This figures shows a distribution of key information of our data from 599 annual reports of 200 firms. Series 1 represents the reports where firms did not report any species and Series 2 documents the reports in which firms included the names of species (we counted the number of species as explained in the data description). REPORTS is the number of reports in our data. FIRMS is the number of firms (during 2012–2016) in each set of Series. AWARD is the number of reports indicating the firms received environment award. ASSURANCE is the number of reports which mention whether the firms receive assurance. BIG 4 is a subset of ASSURANCE indicating whether the assurance received from BIG 4. POP represents the number of reports which mentions the presence of biodiversity/wildlife partnerships

Our results show that 452 out of 599 reports (75%) failed to disclose any species numbers (refer to Fig. 1). These findings support prior literature (Adler et al., 2018; Bhattacharya & Managi, 2013) and suggest a call for awareness among companies (Rimmel & Jonäll, 2013; Hassan et al., 2020b). However, the remaining EC/FT companies, 147 out of 599 (representing 25%), provided species numbers, demonstrating ecological awareness by engaging in conservation efforts to protect and restore species and habitats, valuing species as stakeholders. We found a slight increase in species numbers over years which optimistically displays a deep-ecological view with companies being self-aware of the fundamental value of the planet (Hassan et al., 2020b). The declaration of the SDGs in 2015 may also explain the increase. We acknowledge that this is a small proportion of the sample. However, these EC/FT companies provide seminal knowledge in establishing relationships between species and developing solutions to meet SDGs 14 and 15. These disclosures may be a reporting exercise to manage impressions. However, we expect post COVID-19 that there will be a seismic shift in company reporting and committing to protecting nature, and therefore justify the significance of our results.

Table 2 presents the descriptive statistics for all variables used in the study. The average number of species disclosed by companies is about 3 species with a standard deviation of 21. This implies that there is a significant difference in the reported species among the companies. We further nalysed by country and found developing countries have a higher mean score for the number of species (i.e., 3.458) than developed countries (i.e., 2.614). This supports the recent arguments, where experts believe that developing countries are to blame for the COVID-19 pandemic due to illegal wildlife trade and wet markets and call for enforcing the banning of such trades to prevent further pandemics and extinctions and forcing companies to become socially responsible (Ceballos et al., 2020; Ma et al., 2020). Legitimacy theory explains that developing countries provide more species to maintain societal legitimacy and reputation (Adler et al., 2018; Bhattacharyya & Yang, 2019). We found developed countries have a higher mean score for assurance, environmental award, partnerships, Big4, leverage, governance, industry, and CO_2_ emissions, which is similar to prior studies on CSR (Bouten et al., 2011; Tagesson et al., 2009). Furthermore, the developing countries had a higher mean score for environmental score, firm size, GDP, and inflation which supports the literature that finds a positive relation with CSR disclosure (Chiu & Wang, 2014; Huang & Kung, 2010).

**Table 2:** Summary statistics of the variables used in our models

	Full sample				Developing country		Developed country		Difference in means
	Obs	Mean	Median	St.Dev	Obs	Mean	Obs	Mean	*t*-test
Number of species	599	2.863	0.000	21.552	177	3.458	422	2.614	0.844
Buying assurance	599	0.679	1.000	0.467	177	0.599	422	0.714	− 0.115**
Environment score	599	0.065	0.000	0.250	177	0.170	422	0.021	0.148***
Green	599	0.658	1.000	0.475	177	0.588	422	0.687	− 0.100**
Environment award	599	0.199	0.000	0.399	177	0.186	422	0.204	− 0.018
Presence of partnership	599	0.237	0.000	0.425	177	0.147	422	0.275	− 0.128***
Big 4	599	0.391	0.000	0.488	177	0.367	422	0.401	− 0.033
Firm size	590	12.494	11.809	2.512	171	13.276	419	12.175	1.101***
Log (revenue)	593	11.346	11.234	0.545	174	11.276	419	11.375	− 0.099**
Leverage	596	6840.108	0.383	167,000	174	16.66	422	9653.568	− 9636.907
Governance	599	0.839	1.232	0.785	177	− 0.246	422	1.294	− 1.540***
CO_2_ emission	400	10.426	9.200	4.645	116	7.545	284	11.602	− 4.058***
Log (forest area)	596	13.322	14.527	2.014	174	14.100	422	13.002	1.098***
GDP growth	596	2.820	2.200	2.528	174	5.681	422	1.641	4.040***
Inflation	596	1.552	1.400	1.504	174	2.344	422	1.226	1.118***

Table 3 provides the correlation matrix of all variables included in the study. To identify any multicollinearity problems, we followed prior studies (Bhattacharyya & Yang, 2019; Haniffa & Cooke, 2005) and found a maximum of 0.161 as a correlation coefficient.

**Table 3:** Correlation Matrix (the numbers 1–15 in top row represent the number mentioned for each variable)

Variables	1	2	3	4	5	6	7	8	9	10	11	12	13	14	15
1. Number of species	1.000														
2. Buying assurance	0.029	0.029													
3. Environment score	0.086**	0.086**	0.086**												
4. Green	− 0.017	− 0.017	− 0.017	− 0.017											
5. Environment award	− 0.012	− 0.012	− 0.012	− 0.012	− 0.012										
6. Presence of partnership	0.161***	0.161***	0.161***	0.161***	0.161***	0.161***									
7. Big 4	− 0.004	− 0.004	− 0.004	− 0.004	− 0.004	− 0.004	− 0.004								
8. Firm size	0.011	0.011	0.011	0.011	0.011	0.011	0.011	0.011							
9. Log (revenue)	− 0.005	− 0.005	− 0.005	− 0.005	− 0.005	− 0.005	− 0.005	− 0.005	− 0.005						
10. Leverage	− 0.004	− 0.004	− 0.004	− 0.004	− 0.004	− 0.004	− 0.004	− 0.004	− 0.004	− 0.004					
11. Governance	− 0.024	− 0.024	− 0.024	− 0.024	− 0.024	− 0.024	− 0.024	− 0.024	− 0.024	− 0.024	− 0.024				
12. CO_2_ emission	− 0.013	− 0.013	− 0.013	− 0.013	− 0.013	− 0.013	− 0.013	− 0.013	− 0.013	− 0.013	− 0.013	− 0.013			
13. Log (forest area)	0.026	0.026	0.026	0.026	0.026	0.026	0.026	0.026	0.026	0.026	0.026	0.026	0.026		
14. GDP growth	− 0.067*	− 0.067*	− 0.067*	− 0.067*	− 0.067*	− 0.067*	− 0.067*	− 0.067*	− 0.067*	− 0.067*	− 0.067*	− 0.067*	− 0.067*	− 0.067*	
15. Inflation	0.068*	0.068*	0.068*	0.068*	0.068*	0.068*	0.068*	0.068*	0.068*	0.068*	0.068*	0.068*	0.068*	0.068*	0.068*

The variables are defined in the variable description table

***p < 0.01, **p < 0.05, *p < 0.1

We tested the presence of multicollinearity by calculating the variance inflation factor (VIF) and tolerance values for each of the explanatory and control variables, as presented in Appendix A. The VIF indicates how much the variance of a coefficient is inflated because of the linear dependence with other variables, and the tolerance is the extent of variability of selected regressors not explained by the other regressors. The threshold values for VIF and tolerance are less than 10 and more than 0.1, respectively (see Gujarati, 2003; Hair et al., 2013). Appendix A shows that the VIFs are below 5 (except the Governance variable, which is 5.88). So, multicollinearity is not a problem for our estimations of models.

### Empirical Models

To test our hypotheses, we started with OLS regression by using the natural logarithm of the number of species as our dependent variable. However, the coefficients of the regression led to inconsistent estimators of the parameters of our model. Following Gourieroux et al. (1984), therefore, we used the Poisson pseudo-maximum likelihood (PPML) regression with multi-way fixed effects model, since our dependent variable—the number of species—is a non-negative count variable. In other words, the number of species, a Poisson random variable, has a discrete probability distribution that indicates the probability of a given number of species reported in a fixed interval of time. In related simulation results by Santos Silva and Tenreyro (2011), it shows that the PPML model provides better results even when the dependent variable contains a large number of zeros. So, we believe that the Poisson distribution model is appropriate for our data. This is because 75% of our sample did not provide disclosure on species protection. In general, we write our empirical model as

1 $$\begin{gathered} Y_{{{\text{it}}}} = f({\text{Buying Assurance}},{\text{ Environment Score}},{\text{ Environment Award}},{\text{ Green}},{\text{ Presence of Partnership}}, \hfill \\ {\text{Big 4}},{\text{ Firm- and Country-level control variables}}) \hfill \\ \end{gathered}$$

where, *Y* is the number of all species disclosed on financial reports of sample firm *i* in year *t*.

In particular, for our Poisson model, the conditional mean of *Y* in Eq. (1) is written as $E(Y|X)=\mathrm{exp}(X\beta )$

where *X* is the vector independent variable (shown in Eq. 1) and a constant. The maximum likelihood estimator of $\beta$, the coefficients of relevant independent variables, is calculated by maximizing the following log-likelihood function:

2 $$L\left(\beta \right)= \sum {Y}_{t}\mathrm{log}\left({\alpha }_{t}\right)-{\alpha }_{t}-\mathrm{log}({Y}_{t})$$

where ${\alpha }_{t}=exp({x}_{t}^{^{\prime}}\beta )$ shows a model for the conditional mean of the number of species. However, there may exist a problem with the model as it imposes an assumption of mean–variance equality in its empirical application. Thus, an alternative model could be a negative binomial model. But our outcome variable, i.e., the number of species, seemed unlikely to follow a negative binomial distribution. So, in accordance with Cameron and Trivedi (1990), we tested for over-dispersion. We failed to reject the null hypothesis of mean–variance equality in all the estimations. This justifies the use of Poisson for our sample.

### Empirical Results

In Table 4, we report the results of the Poisson regression, to explain the results for the likelihood of the number of species disclosed by the companies. In addition to the coefficients, we also show White’s heteroscedasticity robust standard errors in parenthesis.^13^ In Model 1 of Table 4, the model is estimated by traditional Poisson regression with year and country fixed effects. In Model 2, we report the Poisson pseudo-maximum likelihood (PPML) regression with multi-way fixed effects.

**Table 4:** Poisson regression results for the likelihood of number of species disclosed

Dependent variable: number of species		
	Poisson regression with year and country FE	PPML with multi-way FE
	(1)	(2)
Buying assurance	1.129**	1.131*
	(0.498)	(0.601)
Presence of partnership	4.093***	4.111***
	(0.446)	(0.477)
Environment Score	1.533*	1.530**
	(0.800)	(0.701)
Environment award	− 1.948***	− 1.937***
	(0.455)	(0.454)
Big 4	− 1.675***	− 1.663***
	(0.552)	(0.543)
Green industry	0.226	0.233
	(0.454)	(1.093)
Firm size	0.059	0.062
	(0.069)	(0.085)
Log (revenue)	− 0.283	− 0.313
	(0.366)	(0.418)
Leverage	− 0.000***	0.020
	(0.000)	(0.025)
Governance	3.991*	3.912**
	(2.193)	(1.865)
CO_2_ emission	− 1.033*	− 1.055
	(0.625)	(0.822)
Log (forest area)	0.727*	0.726**
	(0.384)	(0.283)
GDP growth	− 0.559*	− 0.561**
	(0.315)	(0.274)
Inflation	0.546*	0.546*
	(0.285)	(0.308)
Constant	− 15.481	1.021
	(9.832)	(13.564)
Observations	394	373
Pseudo R^2^	0.776	0.773
Country fixed effects	Yes	Yes
Year fixed effects	Yes	Yes

The table reports the effects of assurance, environment award, presence of partnership and red Industry on the number of reported species in firm’s annual report in each year. The data consists 599 firm-year observations of top 200 firms listed in Global 500 firms for the year 2012, 2014 and 2016. Green industry is a dummy equal to 1 if the industry belongs to green industry and 1 if it is in red or amber industry. Natural logarithm of total assets as a proxy for firm size is used. Model 2 reports Poisson pseudo-maximum likelihood (PPL) regression with multi-way fixed effects. Robust standard errors are clustered at the industry level and reported in parenthesis

***Denotes 1%, **denotes 5% and *denotes 10% significance level

Based on the deep ecology concept, in this paper, we consider species as a stakeholder (Samkin et al., 2014), and therefore, in Hypothesis 1, we propose that there is a positive relationship between the number of species and buying assurance. The proposition in Hypothesis 1 is proven in Models 1 and 2, which show positive and statistically significant coefficients (*Model 1: β* = *1.129, p* < *0.05; Model 2: β* = *1.131, p* < *0.10, respectively*) of buying assurance. It implies that firms with assurance are likely to report a greater number of species than those firms without assurance. Hypothesis 1 reinforces empirical studies which have found that assured information is viewed as more credible and reliable, narrowing the legitimacy gap (Cho et al., 2015a; Maroun, 2018). Theoretically, our results support deep ecology, legitimacy, and stakeholder theories as the overall increase in disclosure optimistically displays a deep-ecological view with companies being aware of the fundamental value of the planet. Our results support Hassan et al. (2020b), which found a positive relationship between assurance and biodiversity disclosures. Theoretically, our results align with deep ecology, evidencing that companies are committed to protecting species (Samkin et al., 2014), and valuing them as stakeholders. Additionally, these findings have implications for policymakers in developing solutions with companies providing assurance on species information. We expect that assurance will become an integral part of companies committing to SDGs 14 and 15. This result is in line with prior studies’ stream of literature that empirically examines the assurance of a company’s CSR report. These studies note that stakeholders place more confidence in CSR reports where the level of assurance provided is reasonably high (Casey & Grenier, 2015; Kolk & Perego, 2010; Mahoney et al., 2013; Peters & Romi, 2015; Pflugrath et al., 2011; Simnett et al., 2009).

In addition, we consider the assurance provider as Big4. Big4 is a binary variable equal to 1 if the sample company has an auditor from one of the Big4 audit firms. The coefficients in Models 1 and 2 show a negative and statistically significant coefficient (*Model 1: β* = *1.675, p* < *0.001; Model 2: β* = *1.663, p* < *0.001, respectively*). This implies the number of species in reporting increases if the company is not audited by one of the big four accounting firms. This additional result confirms that auditors from the Big4 accounting firms may be failing to address the B/E crisis or cannot generate awareness in the company. A deep-ecological concern would expect Big4 providers to relate to the need for uniformed reporting guidelines on species and habitat protection (Atkins & Atkins, 2018). Auditors from the Big4 must regard species as a main stakeholder post-pandemic. To achieve SDGs 14 and 15, assurance providers like Big4 must encourage companies to develop long-term sustainability and their consultancy must include advocating the protection of species environmental recovery.

The coefficients of environment score in Models 1 and 2 are positive and statistically significant (*Model 1: β* = *1.533, p* < *0.10; Model 2: β* = *1.530, p* < *0.05, respectively*). This supports our Hypothesis 2, which states that poor environmental performers are likely to report a greater number of species compared to better environment performers. This result is consistent with prior literature that these companies disclose more to defend legitimacy (Cho et al., 2012; Clarkson et al., 2008). Our results support empirical studies that have found a positive relationship between poor environmental performers and biodiversity disclosure (Hassan et al., 2020b). Theoretically, our results support legitimacy theory, which explains that these poor performers may disclose more species as they negatively impact biodiversity through illegal wildlife trade and lack robust regulations, and therefore, our results may benefit regulators with assisting in preventing further species loss. Deep ecology explains that to achieve SDGs 14 and 15, these poor performers must begin to consider species as a stakeholder to prevent further extinctions. This implies that poor environmental performers are more likely to seek environmental legitimacy and more stakeholders’ satisfaction by reporting useful B/E information.

In Hypothesis 3, we predict that to get an award to narrow the legitimacy gap and prove a deep concern for nature, firms report a higher number of species. But, in Table 4, our empirical result shows an opposite sign in coefficients (*Model 1: β* = *− 1.948, p* < *0.001; Model 2: β* = *− 1.937, p* < *0.001, respectively*). These results are surprising as empirical B/E studies have found a positive relationship with companies gaining an award (Hassan et al., 2020b). Our results do not support prior literature (Adler et al., 2018; Atkins et al., 2014) that companies should showcase conservation efforts in protecting species and calls for further academic examination. Theoretically, this evidence suggests companies are disregarding species as stakeholders and neglecting deep ecology. Such a lack of sensitivity towards B/E will make it difficult in achieving the policymakers’ target of B/E prevention. Our justification for such results is due to lack of awareness and so far, there is no mandatory requirement to disclose on the number of species. This highlights that there is a huge call for awareness for species to be regarded as stakeholders by companies. Furthermore, our results suggest a shift in corporate governance consciousness to embed deep-ecological concern by protecting species and their habitats and to prevent future pandemics like COVID-19. By viewing species as stakeholders post-pandemic, they are responsibly committing to achieving SDGs 14 and 15. Thus, if companies become ecologically conscious of the biodiversity crisis, environmental awards will demonstrate their commitment to sustainable development.

Further, we test the association between the presence of wildlife conservation partnership and number of species. Models 1 and 2 show positive and statistically significant coefficients (*Model 1: β* = *4.093, p* < *0.001; Model 2: β* = *4.111, p* < *0.001, respectively*). This implies that firms which have a wildlife conservation partnership are likely to disclose a greater number of species. This gives evidence in favor of Hypothesis 4. Our results support prior studies (Adler et al., 2018; Boiral & Heras-Saizarbitoria, 2017; Hassan et al., 2020b) and highlight that wildlife partnerships are a key driver of companies protecting species. This also implies additional empirical support for our multi-theoretical perspective that integrates insights from legitimacy, stakeholder, and deep ecology theories. Specifically, deep ecology explains how companies engaging in partnerships are preserving nature (Atkins et al., 2018), and confirms that companies view species as stakeholders by protecting their habitats and realizing their intrinsic worth. Our finding implies that companies respond to increased stakeholder expectation by proactively engaging in comprehensive wildlife conservation partnerships, which leads to better B/E-related activism and B/E reporting. Companies may indeed be showcasing conservation efforts to protect species and habitats to gain legitimacy (Adler et al., 2018). However, partnership engagement increases knowledge and can address the extinction crisis and prevent future pandemics. To meet SDGs 14 and 15, collaboration and shared knowledge are encouraged (Jones & Solomon, 2013). Our results imply that partnership association can help develop solutions (Bebbington & Unerman, 2018; Gibassier et al., 2020) and achieve a sustainable future. This evidence is also in line with our multi-theoretical framework that implies that companies use these mechanisms as public relationships instruments to legitimize their existence (e.g., Adler et al., 2018; Hassan et al., 2020b) and oversee the perceptions of the pertinent stakeholders (Bhattacharyya & Yang, 2019).

Finally, regarding control variables, we find Governance, Forest area, and Inflation are statistically significant and positively related to the number of species, suggesting that country-level variables are key determinants of disclosure on the number of species. Overall, our hypotheses are mostly supported by our empirical findings.

### Robustness Tests

In the previous section, we use Poisson regression to investigate the relationship between company-level variables (such as assurance, presence of partnerships, firm size) to the number of species. In this section, we analyze our data in three phases: (1) dividing the data into two sub-samples, based on the firms headquarter, in developed and developing countries; (2) analyzing the full sample by taking care of over-dispersion of the data by reclassifying the sample into four groups based on the number of species disclosed, and (3) dividing the sample into financial and non-financial companies.

In our first test, we report the results of the variables that influence companies to commit to a greater number of species disclosure and how those variables work among firms operating in developed and developing countries. The results of Poisson regression at the firm-level data for developed and developing countries are reported in Table 5.

**Table 5:** Robustness tests: Poisson regression

Dependent variables	Developed country	Developing country	Full sample (ZIP)
	Poisson regression		Zero-inflated Poisson regression
	Number of species		*nSpecies*
	(1)	(2)	(3)
Buying assurance	0.427	2.314***	0.100*
	(0.541)	(0.560)	(0.059)
Presence of partnership	4.029***	2.765***	0.070**
	(0.466)	(0.431)	(0.030)
Environment score	− 0.486	2914.660***	− 0.109**
	(2.572)	(630.495)	(0.046)
Environment award	− 2.110***	1.854***	0.139**
	(0.685)	(0.469)	(0.062)
Big 4	− 1.867***	− 1.951**	− 0.066
	(0.549)	(0.821)	(0.102)
Green industry	n.a.	n.a.	0.054
			(0.039)
Firm size	0.156**	− 0.743***	− 0.004
	(0.075)	(0.169)	(0.013)
Log (revenue)	− 0.871*	1.481***	− 0.029
	(0.489)	(0.246)	(0.049)
Leverage	− 0.000***	− 0.057***	− 0.001
	(0.000)	(0.015)	(0.001)
Governance	− 6.800	9.205***	0.001
	(6.130)	(2.300)	(0.246)
CO_2_ emission	− 0.588	2.534*	n.a.
	(0.671)	(1.317)	
Log (forest area)	0.399	− 266.271***	0.027
	(0.463)	(58.215)	(0.030)
GDP growth	− 0.193	− 1.078***	− 0.037**
	(0.541)	(0.334)	(0.018)
Inflation	1.268**	− 1.196***	− 0.019
	(0.565)	(0.310)	(0.043)
Constant	9.234	3191.847***	0.005
	(11.970)	(696.727)	(0.420)
Observations	282	112	587
Pseudo R^2^	0.807	0.924	n.a.
Country fixed effects	Yes	Yes	Yes
Year fixed effects	Yes	Yes	Yes

The table reports the effects of assurance, environment award, presence of partnership and green Industry on the number of reported species in firm’s annual report in each year. The data consists 599 firm-year observations of top 200 firms listed in Global 500 firms for the year 2012, 2014 and 2016. *nSpecies*-coded from 0 to 4–0 if the number of species is 0, and 1–4 if the number of species is in the range of 1–99, 100–199, 200–299, 300 and more respectively. Green industry is a dummy equal to 1 if the industry belongs to green industry and 1 if it is in red or amber industry. Natural logarithm of total assets as a proxy for firm size is used. Column 3 reports Zero-inflated Poisson (ZIP) regression. Robust standard errors are reported in parenthesis

***Denotes 1%, **denotes 5% and *denotes 10% significance level

Our findings in Table 5 mostly support our hypotheses. Table 5 reveals that *Environment Score* and *Environment Award* have a significant impact on disclosure of the number of species protected in companies that operate in developing environments compared with their counterparts that operate in developed countries. These findings are supported by the legitimacy theory expectation that companies from developing countries want to portray a positive corporate image and influence stakeholder perception (Hassan et al., 2020b; Tagesson et al., 2009). Similarly, we find that assurance has a significant positive effect on disclosure of the number of species protected in companies that operate in developing environments, compared to their counterparts operating in developed countries. Overall, the results support our claim that institutional context has a moderating effect on the relationship between *Environment Score, Environment Award, Assurance,* and disclosure of the number of species.

In the second test, we reclassified our dependent variable scores into a number of classifications. The first classification includes all companies which scored zero; the second classification contains companies which scored between 1 and 99. The third classification contains companies which scored between 100 and 199, and the fourth classification those which scored between 200 and 299 and more.

Let us consider *Y*_*i*_ an observable variable having discrete numbers 0, 1, 2, 3, and 4 based on the reported number of species. Let ${y}_{i}^{*}$ represent an unobservable variable that captures the level of concerns for biodiversity—proxied by the number of species of *i*th firm. The outcome of diversity can be represented as a function of a vector of explanatory variables (*x*_*i*_) and relevant control variables using the following linear relationship:

3 $${y}_{i}^{*}=\phi {x}_{i}^{^{\prime}}+\delta \sum controls+{u}_{i}, where {u}_{i}\sim N(\mathrm{0,1})$$

where $\phi$ is a vector of unknown parameters. Let us consider ${m}_{i}$ and ${y}_{i}^{*}$ are related to the observable variable ${Y}_{i}$. Consider ${m}_{i}$ as four levels of the reported number of species representing the extent of biodiversity by *i*th firm. ${m}_{i}$ determines five observed values as below:

$$Y_{i} = 0\left( {{\text{`unable}}\;{\text{to}}\;{\text{report}}\;{\text{any}}\;{\text{species'}}} \right)\;{\text{if}}\;y_{i}^{{\text{*}}} = 0;$$

$$Y_{i} = 1\left( {{\text{`less}}\;{\text{than}}\;{\text{satisfactory}}\;{\text{level}}\;{\text{of}}\;{\text{reported}}\;{\text{species'}}} \right)\;{\text{if}}\;{\text{0}} < y_{i}^{*} \le m_{1} ;$$

$$Y_{i} = 2\left( {`{\text{reasonably}}\;{\text{satisfactory}}\;{\text{level}}\;{\text{of}}\;{\text{reported}}\;{\text{species'}}} \right)\;{\text{if}}\;m_{{\text{1}}} < y_{i}^{*} \le m_{{\text{2}}} ;$$

$$Y_{i} = 3\left( {{\text{`satisfactory}}\;{\text{level}}\;{\text{of}}\;{\text{reported}}\;{\text{species'}}} \right)\;{\text{if}}\;m_{2} < y_{i}^{*} \le m_{3} ;$$

$$Y_{i} = 4\left( {{\text{`more}}\;{\text{than}}\;{\text{satisfactory}}\;{\text{level}}\;{\text{of}}\;{\text{reported}}\;{\text{species'}}} \right)\;{\text{if}}\;y_{i}^{*} \ge m_{3}$$

Let *x* denote the vector of explanatory variables that influence the number of reported species y^*^ and which are denoted by the following model:

4 $${y}^{*}={x}^{^{\prime}}\beta +\varepsilon$$

The probability of observed *Y* can be estimated by the ordered Probit model. However, following Lambert (1992) we use a zero-inflated Poisson (ZIP) model to measure the relationship between disclosure on the number of species protected and the rest of the research variables denoted as y*, by each sample firm in the years 2012, 2014, and 2016. The results show that buying assurance, presence of partnership, environment score, and award are in line with the previous findings reported in Table 4. However, the coefficient of Big4 is negative but not statistically significant. Overall, we show that our results are robust with different specifications of estimations.

Further, we present the third test of robustness and divide the sample into financial and non-financial companies (see Table 6). The results show that buying assurance, environmental score, and award from non-financial firms are in line with previous findings.

**Table 6:** Robustness tests: Poisson regression results

	Non-financial firms	Financial firms
	Model 1	Model 2
Dependent variable: number of species		
Buying assurance	2.312***	0.660
	(0.851)	(1.081)
Presence of partnership	0.196	− 1.542*
	(1.052)	(0.838)
Environment score	1.761**	− 18.715***
	(0.782)	(2.575)
Environment award	− 1.628**	− 0.987
	(0.774)	(0.816)
Big 4	− 2.448**	− 0.371
	(1.140)	(0.833)
Green industry	4.216***	n.a
	(1.609)	
All control variables	Included	Included
Constant	7.091	− 8.222
	(8.962)	(9.830)
Observations	394	96
Pseudo R^2^	0.568	0.342
Country fixed effects	Yes	Yes
Year fixed effects	Yes	Yes

The table reports the effects of assurance, environment award, presence of partnership and red Industry on the number of reported species in firm’s annual report in each year. The data consists 599 firm-year observations of top 200 firms listed in Global 500 firms for the year 2012, 2014 and 2016. Green industry is a dummy equal to 1 if the industry belongs to green industry and 1 if it is in red or amber industry. Natural logarithm of total assets as a proxy for firm size is used. All control variables are included in both Models 1 and 2 but not shown and Poisson regression is used. Robust standard errors are clustered at the industry level and reported in parenthesis

***Denotes 1%, **denotes 5% and *denotes 10% significance level

### Addressing Endogeneity

Our study uses a Poisson regression model to support the hypotheses (see Table 4). However, the model is likely to suffer from endogeneity. For instance, our sample firms with stronger corporate governance tend to disproportionally report the name of the species (source of selection bias). In addition, the presence of stronger governance may lead to a higher number of reported species. On the other hand, it is also possible that firms with better awareness of B/E tend to attract and hire more expert directors to the board and improve their corporate governance (source of reverse causality). The same argument is true for the environment award variable. To deal with these issues, we use two different methods. Firstly, we follow Geraci et al. (2018) to estimate our model by two-stage residual inclusion (2SRI). Following Lin et al. (2017), we use an instrumental variable, *sindustry*—a dummy variable equal to 1 if our sample firms belong to environmentally sensitive industries, such as energy, chemical, and materials, and 0 otherwise. We use the endogenous variable environment award as a dependent variable and *sindustry* and other exogenous variables (used in Table 4) as independent variables of a logistic model (first stage). We calculate the residual from this first stage and include this residual in the second stage of the same Poisson model (as in Tables 4 and 5). The results are reported in Column 4 of Table 7.

**Table 7:** Robustness tests: addressing endogeneity

	Negative binomial regression			2SRI
Dependent variable	Number of species			
	(1)	(2)	(3)	(4)
Buying assurance	1.694**			1.456***
	(0.855)			(0.537)
Presence of partnership		3.354***		5.004***
		(0.603)		(0.533)
Environment score			1.488***	4.213***
			(0.526)	(0.936)
Environment award			− 14.229**	− 8.838***
			(5.544)	(1.654)
Big 4	− 0.881	1.056**	0.277	− 1.744***
	(0.757)	(0.522)	(0.510)	(0.513)
Green industry	− 0.044	− 0.445	− 0.398	− 0.037
	(0.642)	(0.424)	(0.720)	(0.413)
Firm size	− 0.276***	− 0.615***	− 0.309**	− 0.114*
	(0.100)	(0.152)	(0.131)	(0.064)
Log (revenue)	0.945**	0.757	0.970*	− 0.563*
	(0.457)	(0.491)	(0.543)	(0.310)
Leverage	0.060	− 0.043	0.610*	− 0.001
	(0.047)	(0.057)	(0.360)	(0.004)
Governance	n.a.	n.a.	n.a.	7.028***
				(1.723)
CO_2_ emission	− 0.155*	− 0.120*	− 0.310	− 1.516***
	(0.084)	(0.072)	(3.036)	(0.236)
Log (forest area)	0.638***	0.182	3.436	1.796***
	(0.246)	(0.303)	(4.688)	(0.342)
GDP growth	− 0.575***	− 0.168	0.081	− 1.220***
	(0.162)	(0.164)	(0.626)	(0.216)
Inflation	− 0.285	− 0.420**	0.115	0.736**
	(0.234)	(0.200)	(0.376)	(0.305)
Residual from 1st stage				3.128***
				(0.661)
Constant	− 13.994**	− 6.036	− 45.575	− 28.562***
	(5.758)	(6.391)	(67.998)	(7.855)
LR Chi^2^	44.90**	59.38***	46.38**	
	(0.016)	(0.000)	(0.017)	
Observations	114	114	114	369
Pseudo R^2^	0.072	0.151	0.194	0.799
Country fixed effects	Yes	Yes	Yes	Yes
Year fixed effects	Yes	Yes	Yes	Yes

The table reports the effects of assurance, environment award, presence of partnership and green Industry on the number of reported species in firm’s annual report in each year. The data consists 599 firm-year observations of top 200 firms listed in Global 500 firms for the year 2012, 2014 and 2016. Green industry is a dummy equal to 1 if the industry belongs to green industry and 1 if it is in red or amber industry. Natural logarithm of total assets as a proxy for firm size is used. Columns (1)–(3) reports Negative Binomial regressions after using propensity score matching. Column (4) represents the 2-Stage Residual Inclusion (2SRI) regression. Robust standard errors are reported in parenthesis

***Denotes 1%, **denotes 5% and *denotes 10% significance level

In the second approach, in accordance with Quigley et al. (2019), we employ a propensity score matching (PSM) procedure to form matched sets of treated and control firms which share a similar value on the propensity score (see Heckman & Todd, 2009). This is also a two-step procedure. We construct a dummy variable *lgovernance* equal to 1 if a firm has a governance score less than the industry average governance score, and 0 otherwise. We run a logistic model to estimate the probability that a firm would buy an external assurance with weaker governance score. This regression generates a propensity score. We follow Dehejia and Wahba (2002) to match each treatment firm to a control firm using the nearest neighbor algorithm with replacement and setting the caliper to 0.25*standard error of the propensity score. Appendix B shows each covariate (control variables reported) after the PSM procedure across treatment and control firms. We re-estimate the matched sample with a negative binomial regression. The results are reported in Columns 1–3 of Table 7.^14^ The likelihood ratio of Chi^2^ test for the joint significance of independent variables is statistically significant and therefore indicates a good model fit. Overall, both the procedures qualitatively support our main results and hypotheses.

### Developing solutions and providing examples from EC/FT companies

From the empirical findings, we suggest how companies can help to achieve SDGs 14 and 15 and lead future reporting in sustainable development. B/E accounting aligns with wider disciplines in CSR research, such as global warming and climate concerns. High on the economic agenda from the COVID-19 recovery is the movement from a vulnerable ‘business as usual’ to a sustainable ‘green recovery’, rebalancing the human relationship with nature (Porritt, 2020). Failure to provide sustainable solutions, and if efforts to achieve the wider UN goals, including SDGs 14 and 15 are not met, the planet could be heading for an apocalyptic crisis. We believe that due to the urgency of the wider global environmental challenges facing humanity (Sobkowiak et al., 2020), developing solutions is a matter of urgency. We anticipate post COVID-19, biodiversity and species accountability will be a core element of corporate reporting. Companies must be responsible for committing to achieving SDGs 14 and 15, as a failure to protect and conserve nature will have a catastrophic financial impact (WEF, 2020). Our results indicate that pre-COVID-19, only 25% of companies embedded an in-depth ecological perspective and valued species as stakeholders, which can explain positive relations with species numbers and companies gaining assurance and partnership engagement. Furthermore, our results indicate there must be a seismic shift from poor environmental performers and companies who wish to gain awards, to consider species as stakeholders, and align with global sustainability. Inspired by SDGs 14 and 15, we explain that if companies collectively implement B/E reporting, this may offer solutions for a sustainable society. As such, rethinking accounting frameworks and guidelines is needed (SER, 2016), and accounting professionals need to know how they can incorporate the B/E into their activities. For instance, Royal Dutch Shell displays responsible disclosure:

“When we operate in critical habitats—those that are rich in biodiversity and important to conservation—we apply stringent mitigation standards. We were also the first in the energy industry to introduce a biodiversity standard.” (Royal Dutch Shell, company website).

This demonstrates how companies can regard species as stakeholders by transparently disclosing their efforts for nature to flourish. For example, Volkswagen has stated its efforts in protecting and restoring biodiversity and species as follows:

“In the grounds of the Volkswagen factory in Emden, a colony of bees on the verge of extinction (Apis millifica) has been successfully established, growing from 5000 to 40,000 bees in just a short space of time. Plans are underway to plant a further 1000 fruit trees to ensure a plentiful supply of food for the bees.” (Volkswagen, CSR report, 2014).

## Summary and Conclusion

The main aim of this paper is to explore the disclosure of the number of species from the top 200 Fortune Global companies to understand how EC/FT companies are answering the collapse of biodiversity and threat of further species extinction. We create and test with Poisson pseudo-maximum likelihood (PPML) regression, the relationship between the number of species protected and its determinant factors. Using a comprehensive sample of Fortune Global companies from before the COVID-19 pandemic, from 2012, 2014, and 2016, our results reveal that 75% of reports omit any species numbers, showing that most companies are failing to address the B/E crisis. However, the remaining 25% present how EC/FT companies have initiated efforts before the current pandemic, which can influence companies’ reporting in the post COVID-19 era. We contribute to developing solutions with this empirical study by extending B/E literature and encouraging companies to provide responsible reporting and disclose B/E accountability to align with SDGs 14 and 15.

**Fig. 2:**
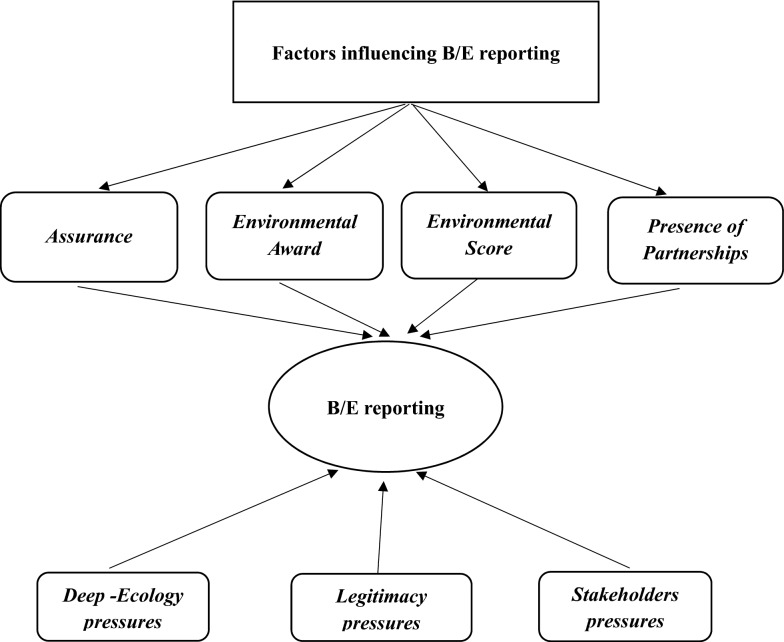
Factors influencing B/E reporting

The empirical model is based on a comprehensive theoretical framework. These findings support prior B/E disclosure studies that have found that few companies provide B/E information (Adler et al., 2018; Hassan et al., 2020b). This evidences a huge call for companies to make transformational changes from anthropocentrism, given the urgency of the extinction crisis and existential threat to civilization (Ceballos et al., 2020). The link to COVID-19 and human infringement with nature through illegal wildlife markets and trade is inextricably linked to business survival. Corporates are dependent on natural resources and must cease to be disrespectful to nature and engage in stewardship of natural assets (Jones, 1996). However, we focus on the remaining EC/FT companies (25%) that are responding to the extinction crisis and showing efforts to conserve and protect habitats. Supporting deep ecology theory and recognizing species as stakeholders, these companies are displaying responsible corporate governance by embedding deep ecological culture. Specifically, our regression presents significant relationships between the number of species disclosed and assurance, poor environmental performers, presence of partnerships, and gaining environmental awards (refer to Fig. 2). Our results imply that there is a huge call for corporate consciousness and opportunity for companies to display their responsible efforts.

These findings make an important contribution to B/E literature in at least four ways. First, our results suggest assurance for B/E reporting as one important channel through which corporate governance may influence B/E outcomes, ascertaining a clear mechanism through which external corporate governance may influence the firm’s B/E reporting. Second, to our knowledge, this study is the first to directly measure the number of species using a comprehensive dataset. Thus, we can demonstrate a clear positive relationship between environmental performance (incentives for species protection) and species reporting, contributing to the nascent but growing research investigating how environmental performance and species reporting activities might be associated. Third, we contribute more broadly to the literature by improving the understanding of the antecedents of species reporting of firms using a cross-country sample. Fourth, this study extends the extant B/E literature by taking species reporting into consideration in a country-level institutional context. It has been argued in the accounting literature that developing economies suggest an interesting setting that explores both regular questions in addition to a unique phenomenon (Lau et al., 2016). Last, we suggest a reporting strategy for companies to achieve wider global efforts of achieving SDGs 14 and 15 by advocating B/E accountability. Our evidence shows that institutional context has a moderating effect on the relationship between environment score, environment award, assurance, and disclosure of the number of species.

These results have several implications. Our evidence implies that businesses should begin to realize the intrinsic worth of natural capital and their reliance on balanced ecosystems to survive. Embedding ecological culture and aligning with societal and government expectation to prevent further pandemics will be crucial for future organizational survival. Additionally, our findings suggest that companies who engage with wildlife partnerships are leading in the protection of species. These results imply that partnerships are a key driver in preventing further species extinctions and preventing pandemics. The collaboration between wildlife and biodiversity experts and companies can achieve long-term sustainability and contribute to developing solutions. Our work can also guide policymakers in developed and developing nations to the need to improve the surveillance and capacity of the regulatory frameworks in the context of B/E reporting, and to improve stakeholders’ pressures to enhance B/E reporting adoption. Specifically, policymakers and regulators need to make a generally agreed set of B/E reporting guidelines and assurance standards. Collectively, the visionary SDGs can be met by the targeted 2030, as failure to meet these targets will have severe consequences to societal health and economic systems. This research aligns with the United Nations initiatives and post 2020 biodiversity frameworks and will guide decision-makers to understand how disclosure of species protection by companies can assist society to mitigate such risks in the future.

One of the caveats of this study is that our sample is a qualitatively small representation of a larger population to which it could apply. Our sample selection is limited to the top 200 companies from the Fortune Global. Therefore, future studies could extend the number of companies or countries. Second, counting species numerically on its own is not enough to advance solutions. Although this research provides a seminal understanding of the relationship between species and key determinants, additional future research might qualitatively examine corporate motivations and emotions for such disclosure, in order to support our findings. Third, future studies might consider case-studies and interviews with executives, boards, shareholders, and stakeholders to investigate their views on corporate initiatives to protect species and biodiversity, and to align with the SDGs. There is a distinct lack of primary data analysis in B/E literature; this would provide invaluable insights into company motivations to protect species. Also, there are undoubtedly other factors affecting the number of species disclosed, such as ethnic identity, religion, country-level or industry-specific factors, which requires an expanded dataset to consider these additional indicators. Research on why and how companies’ engagement in biodiversity loss and species extinction reporting differs across nations is a promising avenue for future work. In addition, exploring the influence of ethical decision making on biodiversity loss and species extinction reporting across cultures would be a fruitful avenue for further work. Furthermore, a potential avenue for future research is to investigate the alignment of B/E, circular economy model, and integrated reporting to enhance the development of solutions for a sustainable society.

## Appendix A

Variance inflation factor (VIF)

**Table:**

Variable	VIF	Tolerance
Number of species	1.04	0.9593
Buying assurance	1.56	0.6396
Environment score	1.29	0.7755
Green	1.19	0.8397
Environment award	1.14	0.8799
Presence of partnership	1.27	0.7853
Big 4	1.51	0.6639
Firm size	1.19	0.8402
Log (revenue)	1.20	0.8306
Leverage	1.03	0.9668
Governance	5.88	0.1701
CO_2_ emission	2.91	0.3442
Log (forest area)	2.96	0.3381
GDP growth	3.24	0.3083
Inflation	1.79	0.5586

## Appendix B

Covariate balance of control variables

**Table:**

Variables	Unmatched (U)/Matched (M)	Treated		Control	*t*-test
Big 4	U	0.379		0.395	− 0.36
	M	0.404		0.421	− 0.19
Green industry	U	0.310		0.800	− 12.96***
	M	0.632		0.596	0.38
Firm size	U	12.304		12.569	− 1.16
	M	12.494		12.728	− 0.51
Log (revenue)	U	11.516		11.277	4.95***
	M	11.294		11.376	− 0.89
Leverage	U	2.089		9591.4	− 0.63
	M	1.570		1.429	0.09
CO_2_ emission	U	9.501		10.808	− 2.58**
	M	11.182		10.663	0.58
Log (forest area)	U	13.450		13.269	0.99
	M	13.478		13.393	0.21
GDP growth	U	3.662		2.473	5.33***
	M	3.366		2.794	1.18
Inflation	U	1.7603		1.466	2.18
	M	1.3614		1.603	− 1.08

Firms with low governance and high governance score for unmatched and matched samples.

***Denotes 1%, **denotes 5% and *denotes 10% significance level.

## Footnotes

1 In this paper, we refer to accountability as companies’ disclosure on how to protect species to eliminate biodiversity loss in the future.

2 We identify species as the variety of plants (flora) and animals (fauna) that provide balanced ecosystems essential for human survival and welfare (Sandifer et al., 2015).

3 We refer to the United Nations Sustainable Development Goals (SDGs) 14 *‘Life below water*—Conserve and sustainably use the oceans, seas and marine resources for sustainable development’ and SDG 15 ‘Life *on Land*—Protect, restore and promote sustainable use of terrestrial ecosystems, sustainably manage forests, combat desertification and halt and reverse land degradation and halt biodiversity loss’.

4 We define biodiversity loss as the decline of the infrastructure of ecosystems caused by the abuse from human and business overexploitation (Hassan et al., 2020a; Roberts et al., 2020a).

5 We define ecologically conscious or forward-thinking companies as those companies that realize the importance of biodiversity and species extinction and have provided disclosure on how to protect both biodiversity and species before the COVID-19 crisis. Therefore, we use ecologically conscious and forward-thinking companies interchangeably.

6 The recent Intergovernmental Science-Policy Platform and Ecosystem Services Global Assessment Report (IPBES, 2019) outlines one million species which are under threat from extinction within decades if transformational changes are not made. Such species decline challenges organizational viability as they are dependent on healthy ecosystems providing natural assets for sustainable economic activity (European Business and Biodiversity Campaign, 2020). Although no monetary value can be placed on natural assets (Jones & Solomon, 2013), the value ecosystems provide in resources is estimated to be between $125 to $145 trillion per year (Costanza et al., 2014), highlighting the intrinsic worth of natural resources to businesses.

7 For example, crop production which has an estimated annual valuation of $577 billion, is under threat from pollination loss and bee decline (The World Bank, 2020), threatening the food industry. Approximately 70% of cancer drugs are derived from plant species, consequently threatening the pharmaceutical industry (PWC & WWF, 2020).

8 Big4—one of the big four accounting firms: Deloitte, Ernst & Young, KPMG, PwC.

9 The 28 keywords are "Extinct", "Extinction", "EN11", "EN12", ‘EN13", "EN14", "Wildlife", "Habitat", "Species", "Biodiversity", "Biodiversity offset", "Forest", "Ecosystem", "Flora", "Fauna", "Endangered", "Threatened", "Vulnerable", "Accident" (relating to B/E), "Conservation", "Biological diversity", "Protected", "Floral/Faunal wealth", "Rehabilitation", "Groundwater", "Marine", "Vegetation", "Wetlands" (Adler et al., 2018).

10 Countries are Australia (9), Brazil (12), China (120), France (45), Germany (48), India (3), Italy (12), Japan (57), Luxembourg (3), Malaysia (3), Mexico (6), Netherlands (12), Norway (3), Russia (12), Singapore (3), South Korea (15), Spain (6), Switzerland (15), Taiwan (3), Thailand (3), United Kingdom (24), and USA (186).

11 The 19 sectors are Aerospace (21), Apparel (3), Chemicals (6), Energy (99), Construction (18), Financial (144), Food & Beverage and Tobacco (15), Food & Drugs (33), Health care (30), Household products (6), Industrial (18), Materials (12), Media (3), Motor Vehicles and parts (48), Retails (21), Technology (42), Telecommunication (33), Transportation (15), and Wholesalers (33).

12 High-risk sectors (red zone) are Construction & Building Materials, Electricity, Food & Drug Retailers, Food Producers & Processors, Forestry & Paper, Leisure & Hotels, Mining, Oil & Gas, and Utilities. Medium risk sectors (amber zone) are Beverages, Chemicals, Financial Services, General Retailers, Household Goods & Textiles, Personal Care & Household Products, Pharmaceuticals & Biotech, Support Services, Tobacco, and Transport. Low risk sectors (green zone) are Aerospace & Defence, Automobiles & Parts, Diversified Industrials, Electronic & Electrical Equipment, Engineering & Machinery, Health, Information Technology Hardware, Media & Entertainment, Software & Computer Services, Steel & Other Metals, and Telecom Services.

13 To adjust for the differences between variance and mean score, we use a bootstrapping method in the Poisson regression using 500 replications (not reported). The resampling procedure with replacement creates simulated datasets for the estimation. Our results remain qualitatively similar.

14 We use Pearson Chi^2^ test for observed and expected count frequencies over time and we find that our results are statistically significant at 5% level. This means, for instance, there is a significant relationship between number of species and presence of partnership (or environment award) Chi^2^ (20) = 165.014, obs-599, *p* < 0.00 (or Chi^2^ (20) = 61.72, obs-599,  *p* < 0.00).
